# Incidental Detection of *Onchocerca* Microfilariae in Donkeys (*Equus asinus*) in Italy: Report of Four Cases

**DOI:** 10.3389/fvets.2020.569916

**Published:** 2020-11-06

**Authors:** Roberto Amerigo Papini, George Lubas, Micaela Sgorbini

**Affiliations:** Department of Veterinary Sciences, Veterinary Teaching Hospital “Mario Modenato”, University of Pisa, Pisa, Italy

**Keywords:** microfilariae, *Onchocerca*, onchocerciasis, donkey, *Equus asinus*, Italy

## Abstract

This paper reports the occurrence for the first time in Italy of autochthonous *Onchocerca* infection in donkeys. Four jennies, bred on the same farm, were referred to the Veterinary Teaching Hospital of Pisa for a check-up on ovarian activity (*n* = 3) or for veterinary support during the delivery (*n* = 1). Microfilariae were incidentally detected during the blood smear examination of one jenny. Peripheral blood samples were then collected from the other three jennies and the presence of microfilariae was investigated by Knott's test. Circulating unsheathed microfilariae were identified in all the animals. The level of microfilaraemia was between 1 and 31 microfilariae in 2 mL of blood. Hematological changes showed moderate eosinophilia in one case or both remarkable eosinophilia and basophilia in another case. Based on molecular findings by PCR and sequencing, the microfilariae showed 98% sequence similarity with *Onchocerca* sp. in the NCBI GenBank database (Accession No.: MK541848.1). The present report provides evidence that *Onchocerca* is an etiological agent of parasitic infection in donkeys in Italy. Our findings highlight the importance of screening in donkeys for *Onchocerca* even in the absence of clinical indications.

## Introduction

*Onchocerca cervicalis, Onchocerca reticulata*, and *Onchocerca raillieti* (Onchocercidae, Spirurida) are filarial thread-like nematodes. *O. cervicalis* and *O. reticulata* infect equines worldwide (1). *O. cervicalis* adult females (50–70 cm long) and males (7–10 cm long) live in the ligamentum nuchae. *O. reticulata* adult worms can measure up to 75 cm (females) or 15–20 cm (males) and inhabit the flexor tendons and suspensory ligament of the fetlock (2). *O. raillietti* has only been reported in donkeys in Africa (1) and adult worms live in the ligamentum nuchae (3). They are all viviparous. Adult females release microfilariae (L1) measuring 190–310 μm for *O. cervicalis*, 330–370 μm for *O. reticulata* (4), and 180–217 μm for *O. raillieti* (3). When microfilariae are released by the females, they migrate through connective tissues and accumulate in the lymphatics of the upper dermis (1, 2). The highest concentrations can be found along the linea alba in the ventral midline, especially near the umbilicus (1, 3, 4). Microfilariae are also commonly found in the dermis of the face, neck, withers, thorax, and eyes (1). *O. raillietti* microfilariae have also occasionally been found in the dermis of the head, back, hips, forelimbs, hind limbs, and perineum, though to a limited extent (3). Information is only available for the life cycle of *O. cervicalis* (4), however the transmission pathways of *O. cervicalis, O. reticulata*, and *O. raillieti* are likely to be similar. These parasites are all transmitted by blood-sucking midges of *Culicoides* species (1, 2, 4, 5). Mosquitoes (*Aedes aegypti*) also act as intermediate hosts for *O. cervicalis* (6). Microfilariae are ingested by insect vectors while feeding on an infected animal. After development within the intermediate host to reach the infective third-stage, L3 larvae are transmitted to other susceptible definitive hosts at the subsequent blood meal (4).

Many studies have been conducted on epidemiological, clinical, diagnostic, pathological, and therapeutic aspects of *Onchocerca* infection in horses as reviewed by Dagnaw et al. (1). However, with the exception of a relatively recent case report in Italy (7), all these studies are dated. Moreover, only a limited number of studies have investigated the parasitosis in donkeys, almost all of the published studies in donkeys were from African countries, and most focus on *O. raillieti* (3, 5, 8–10). Currently, little or nothing is known about the current spread of *Onchocerca* infections in donkeys in Italy. In order to fill this gap, we report a case of autochthonous infection in jennies. We discuss various epidemiological and clinical aspects of the infection in this host species.

## Materials and Methods

### Case Presentation and Blood Collection

In June 2019, four adult, pluriparous jennies of the Amiatina breed with ages ranging from 6 to 14 years (median age 13 years) were presented to the Veterinary Teaching Hospital (VTH) of the University of Pisa (geographical coordinates: 43°40′48″N 10°20′55″E). The jennies were from a donkey farm located in central Italy, where animals are used for milk production and reared outdoor in a free animal housing system. On the farm, water *ad libitum* and food were available in shaded resting spots.

Three out of the four were barren jennies admitted to the VTH for an examination of ovarian activity. The other jenny was pregnant and was diagnosed with rupture of the prepubic tendon before pregnancy, thus this jenny needed veterinary care at the time of delivery.

When admitted to the VTH, jennies were housed in collective paddocks 24 h a day, fed with meadow hay *ad libitum* along with commercial equine feed (Equifioc^®^, Molitoria Val di Serchio, Italy) according to the NCR energy recommendations (National Research Council, NRC, 2007), as well as having free access to clean and fresh water.

Upon admission to the VTH, all jennies underwent a complete clinical assessment and coprological examinations by means of a flotation technique with saturated solution of NaCl (specific gravity = 1.20).

The pregnant jenny showed signs of colic 2 days after delivery and thus underwent further clinical investigations. A complete blood count and biochemistry analyses were also performed. For this purpose, a venous blood sample was drawn via jugular venipuncture and collected in two sterile 10 mL vacutainer tubes, one with K3-EDTA and one plain tube for serum harvesting. As soon as the blood collection was completed, the sample was labeled (jenny I), immediately transferred to the laboratory of veterinary clinical pathology, and examined. Likewise, a blood work was also carried out in the three barren jennies from the same donkey farm. Blood collection was performed in the same way as previously described. The samples were labeled for animal identification (jennies II–IV).

### Blood Analysis

Complete blood counts were performed using a combined laser-impedance cell counter (ProCyte®, Idexx Laboratories, Milan, Italy) including red blood cell count, hematocrit, hemoglobin, mean corpuscular volume, mean corpuscular hemoglobin, mean corpuscular hemoglobin concentration, **r**ed blood cell distribution width, total leukocyte count, absolute values of neutrophils, lymphocytes, monocytes, eosinophils, and basophils, platelet count, and mean platelet volume. In order to complete the information on the morphological aspects of red blood cells, white blood cells, and platelets, and to assess the leukocyte differential counts in comparison to the results provided by the cell counter, fresh blood smears were prepared and stained with May-Grundwald-Giemsa (MGG) using an automatic stainer (Wescor Aerospray 7150^®^, Delcon, Milan, Italy). Using bright-field light microscopy, blood smears were screened at 20× and then examined at 100× by a trained clinical pathologist.

The serum biochemical assays were performed on a combined spectrophotometric-immunoturbidimetric device (Analyzer SAT 450^®^, Medical System, Guidonia, Rome, Italy) including: total plasma protein, albumin, gamma glutamyl transferase, aspartate aminotransferase, creatine phosphokinase, blood urea nitrogen, creatinine, calcium, phosphate, total bilirubin, and direct bilirubin. In addition, few electrolytes such as sodium, potassium and chloride were assessed with an ion-selective electrodes device (Electrolyte Analyzer GE200^®^, Medical System, Guidonia, Rome, Italy).

The blood cell counter was set up to evaluate the parameters as equine species. All the reference intervals (RIs) used were derived from an internal assessment carried out by the veterinary clinical pathology laboratory with multi-year experience in examining donkey blood.

### Detection of Circulating Microfilariae

**Figure 1:**
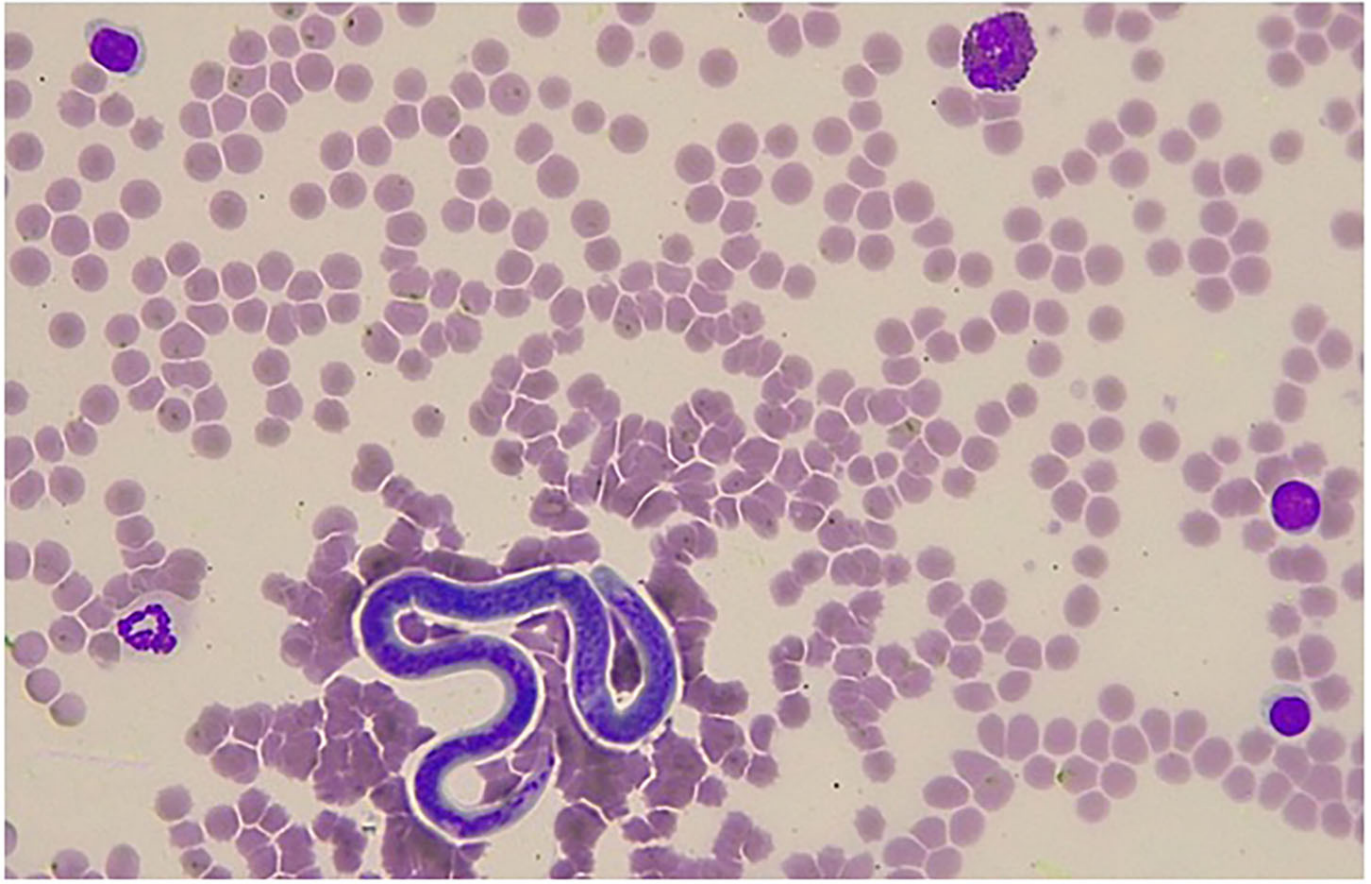
Giemsa stained blood smear showing a single microfilaria of *Onchoerca* sp. detected in a jenny in Italy. In the microscopic field, one eosinophil with characteristic feature of the asinine species, three lymphocytes, and one neutrophil can also be identified (×400 magnification).

Since microfilariae were detected through the blood smear examination of jenny II (Figure 1), the occurrence of peripherally-circulating microfilariae was also evaluated in the other three jennies. Microfilariae detection was performed using a modified Knott's test on peripheral EDTA blood samples collected, as described above. Briefly, 8 mL of distilled water were added to 2 mL of whole EDTA blood and mixed thoroughly. Following centrifugation at 3,000 rpm for 5 min, the supernatant was discarded. The sediment was re-suspended in two drops of distilled water, removed with a 1 mL Pasteur pipette, and examined with a light microscope at 100× magnification. The number of microfilariae was counted in the Knott's test sediment. See the Results section for details on morphometric features. After microscopic examination, another aliquot (2 mL) of EDTA blood sample from each positive jenny was stored at −20°C prior to shipment to a sequencing service provider.

### Molecular Procedures

Molecular analyses were performed by BMR Genomics (Padua, Italy). After thawing at room temperature, genomic DNA was extracted from positive blood samples using the commercial DNA IQ™ System^®^ (Promega) based on paramagnetic beads, following the manufacturer's instructions. A fragment (about 700 base pairs in length) of the mitochondrial gene for cytochrome c oxidase subunit 1 (COX1) was used as a DNA barcoding system and amplified. The PCR amplification was carried out in a final mixture containing 12.5 μL of Master Mix 2X (GoTaq^®^ Green Master Mix, Promega), 2 μL of genomic DNA extracted from blood, 1 μL (10 μM) of forward primer (5′-TGATTGGTGGTTTTGGTAA-3′), 1 μL (10 μM) of reverse primer (5′-ATAAGTACGAGTATCAATATC-3′), and deionized water, reaching a total reaction volume of 25 μL. The PCR reactions were subjected to the following conditions in thermal cycler (Mastercycler^®^, Eppendorf): 95°C × 2 min, then 5 cycles (95°C × 40 s, 45°C for 1.5 min, and 72°C for 1.5 min), followed by 35 cycles (95°C for 40 s, 50°C for 1.5 min, and 72°C for 1 min), and finally 72°C for 10 min. The amplification products were visualized after electrophoresis on 1.5% agarose gel. PCR products were purified by AMPure XP Beads (Beckman Coulter), sequenced, and aligned via BLAST analysis to detect their identity by retrieving similar sequences deposited in NCBI's GenBank database (11, 12).

### Ethics Approval for the Study

Ethical review was not required as per institutional guidelines/local legislation due to natural infections that occurred spontaneously. Written informed consent was obtained from the owners for the participation of their animals in this study.

## Results

All four jennies were apparently healthy on the basis of history and physical examination at admission to the VTH. Three of them (II, III, and IV) remained in this condition throughout hospitalization. However, one jenny (I) was apparently healthy at admission to the VTH but presented signs of colic 2 days after delivering her foal. The results of the complete blood count for all four jennies are reported in Table 1. In jenny I, the values evidenced an anemic condition with normocytic hyperchromic red blood cells. Hyperchromia was due the lipemic sample collected, which interferes with the correct spectrophotometric reading of hemoglobin and induces a high value of mean corpuscular hemoglobin concentration. In the leukogram, neutrophilia, and monocytosis were found along with lymphopenia, all three conditions could be related to a long-lasting inflammatory process or simply related to stress. In jennies II and III, the values did not reveal anything particular in the red blood cell compartment. In the leukogram, eosinophilia was found in both animals and in jenny III it was substantial as was the occurrence of basophilia. In jenny IV, the results of the complete blood count were all within the RIs.

**Table 1:** Alterations detected in the complete blood count of four jennies found incidentally infected with *Onchocerca* microfilariae.

**Hematological values**					Reference range^*^
	**I**	**II**	**III**	**IV**	
Red blood cells M/μL	*3.40*↓	6.47	5.42	5.25	4.4–7.1
Hematocrit %	18.5	35.2	30.9	29.8	27–42
Hemoglobin g/dL	*7.1*↓	12.1	10.9	10.5	9.1–14.7
Mean corpuscular volume fL	54.4	54.4	57.0	56.8	53–67
Mean corpuscular hemoglobin pg	20.9	18.7	20.1	20.0	17.6–23.1
Mean corpuscular hemoglobin concentration g/dL	*38.4*↑	34.4	35.3	35.2	32–37.5
Red blood cell distribution width %	21.4	*22.9*↑	20.5	22.4	16.1–22.5
Total leukocytes K/μL	14.78	10.95	15.69	6.40	6.2–16
Neutrophils K/μL	*11.82*↑	5.04	5.49	3.07	2.4–6.3
Lymphocytes K/μL	*1.18*↓	4.27	6.12	2.28	1.6–8.5
Monocytes K/μL	*1.77*↑	0.33	0.31	0.32	0.0–0.8
Eosinophils K/μL	0.00	*1.31*↑	*3.45*↑	0.73	0.1–1.0
Basophils K/μL	0.00	0.00	*0.31*↑	0.00	0.0–0.2
Platelets K/μL	296	190	295	190	95–360
Mean platelet volume fL	10.4	10.0	10.4	10.1	8.8–12.0
Appearance of plasma	Lipemia ++	Clear	Clear	Clear	NA
Microfilaraemia at the blood smear	0	2	0	0	NA
Microfilaraemia by Knott's technique	4	31	1	3	NA

* *Reference range used internally at the Clinical Pathology Laboratory; the values out-the-range are in italics; ↓, decreased values; ↑, increased values; NA, not applicable*.

The biochemistry analysis in jenny I revealed renal insufficiency highlighted by an increase in both urea (48 mg/dL, RI = 9–31) and creatinine (2.2 mg/dL, RI = 0.59–1.3) as well as a decrease in total proteins (5.6 g/dL, RI = 5.8–7.6) due to a mild reduction in albumin (2.2 g/dL, RI = 2.5–3.2). Both the cholesterol (195 mg/dl, RI = 55–115) and triglycerides (663 mg/dl, RI = 53–248) showed high values. All the other parameters tested, namely calcium, phosphate, gamma glutamyl transferase, aspartate aminotransferase, creatine phosphokinase, total bilirubin, and direct bilirubin were within the RIs. In the electrolytes, sodium (125 mEq/L, RI = 128–138) and potassium (1.6 mEq/L, RI = 3.2–5.1) were low probably due to the interference of the lipemic sample, while chloride was in the normal range. Overall, the trend of these parameters along with the complete blood count results highlighted lipidosis due to a negative energy intake balance related to obstructive colic in a lactating jenny. The clinical symptoms were depression, anorexia, and oliguria (the jenny refused to drink). The mild increase in urea could be related both to anorexia, or along with high levels of creatinine, to an acute pre-renal insufficiency secondary to low ingestion of water. In the other three jennies all parameters on serum biochemistry and electrolytes investigated were within the RIs (values not reported).

In jenny II, microfilaraemia was detected when the blood smear examination by conventional staining and optical microscopy reading was performed by an expert clinical pathologist (Figure 1). On the other hand, no microfilariae were detected through the blood smear evaluation in the other three jennies. MGG stained microfilariae had a wider and rounded anterior end, while the posterior end showed a short, thin, and sharply pointed tail. The microfilariae measured ~214–229 μm in length and 4–5 μm in width. The most prominent feature was the lack of a faintly stained anterior and posterior sheath. This suggested that on the basis of morphological features, they could be differentiated from the sheathed microfilariae of *Setaria* spp and belonged to the genus *Onchocerca*, in which the length of microfilariae between the different species parasitizing donkeys falls within the range of 180–370 μm (3, 4).

Since circulating microfilariae were incidentally detected by microscopic examination of a blood smear from one of the jennies, we thus examined the peripheral blood samples from each jenny by a modified Knott's test, as described above. The Knott's test results showed that all the four jennies presented microfilariae in their peripheral blood samples. Circulating microfilariae appeared to be morphologically identical to each other and from one jenny to another. They were actively motile with serpentine movements, showing the lack of a sac-like hyaline sheath at both ends and a short tail, which matched the characteristics exhibited by the microfilariae in MGG stained blood smears. The characteristic features of the microfilariae appeared to be consistent with *Onchocerca* microfilariae and no further morphometric investigations were carried out, as microfilariae found in Knott's test sediments and in MGG stained smears appeared to be morphologically identical. However, the unusual site of the finding (i.e., peripheral blood stream) precluded any definitive conclusion and only a presumptive diagnosis of asymptomatic onchocerciasis could be made at that time. The levels of microfilaraemia were 31, 4, 3, and 1 microfilariae in 2 mL of blood samples from jennies II, I, IV, and III, respectively. The highest level of microfilaraemia was detected in jenny II which had been found positive from the blood smear. This suggests that positivity at the blood smear was probably related to the higher burden of circulating microfilariae. Table 1 shows the Knott's test results.

The coprological results were positive for gastrointestinal strongyle eggs.

The BLAST search results showed that COX1 of microfilariae from the four examined jennies had the closest sequence similarity (i.e., 98%) with that of *Onchocerca* sp. available from GenBank (Accession Number MK541848.1). Sequences producing significant alignments are shown in Table 2.

**Table 2:** Accession numbers and description of sequences retrieved from GenBank producing significant alignments with COX1 mitochondrial gene of microfilariae incidentally found in peripheral blood samples of four jennies in Italy.

**Accession no**.	**Parasitic organism identification**	**Bit score**	***E*-value**	**Max identity**
MK541848.1	*Onchocerca* sp. JJP-2019	1,138	0.0	98%
KX898458.1	*Onchocerca boehmi* Supperer, 1934	835	0.0	90%
LC318284.1	*Onchocerca flexuosa*	833	0.0	90%
AJ271616.1	*Onchocerca gibsoni*	833	0.0	90%
AM749269.1	*Onchocerca skrjabini*	824	0.0	91%
AP017692.1	*Onchocerca flexuosa*	817	0.0	89%
KX265050.1	*Dirofilaria* sp. “hongkongensis”	817	0.0	89%
AM749270.1	*Onchocerca skrjabini*	813	0.0	91%
AB518693.1	*Onchocerca* sp. wild boar	808	0.0	89%
KX853323.1	*Onchocerca boehmi* Supperer, 1934	806	0.0	90%

Unfortunately, no follow-up was possible as the jenny with the ruptured prepubic tendon died after delivery, while the others returned to the farm a few days after check-up on ovarian activity. The owner was unwilling either to take them back to the VHT for further examination or to allow us to visit his farm at a later time.

## Discussion

To the best of our knowledge, this is the first report of *Onchocerca* infection in donkeys in Italy. The presence of microfilariae in peripheral blood samples was ascertained by laboratory and molecular findings. Unfortunately, the species of microfilariae was not identified. The two *Onchocerca* species infecting donkeys in Europe are *O. cervicalis* and *O. reticulata*, which are both found worldwide (1). The geographical range of *O. raillieti* infection in donkeys known to date is restricted to some countries in Africa (3, 5, 8). In previous studies, when the occurrence of *O. cervicalis* and *O. reticulata* was investigated concurrently, *O. cervicalis* was more prevalent than *O. reticulata*. In fact, prevalence values of 82.98 and 4.26% have been reported for *O. cervicalis* and *O. reticulata* in donkeys, respectively (13). Similarly, prevalence values of 25.42, 5.93, and 2.54% have been reported for *O. cervicalis, O. reticulata*, or both infections in horses, respectively (14). Therefore, based on the results of previous surveys, we believe that *O. cervicalis* was most likely the species involved in our study.

All the four *Onchocerca* infected jennies were bred on the same farm and were kept under the same management conditions. They had always lived on the same farm since their birth and there were no movements throughout the country except for their transportation to the VTH. *Onchocerca* infection in horses is considered rare when macrocyclic lactones are used regularly (15), since treatments with ivermectin (16, 17), moxidectin (18, 19) or doramectin (9) are effective in killing microfilariae (but not against adult worms located in the nuchal ligament). However, since our four jennies were milk-producing animals, no treatment for ectoparasite or endoparasite control had never been performed at the donkey farm in order to prevent the risk of contamination with drug residues in milk intended for human consumption. Moreover, the *Culicoides* species, which may act as intermediate hosts for *Onchocerca*, are spread throughout Italy, including central regions (20) where the farm of the infected jennies is located. Therefore, the examined jennies likely acquired the infection locally on the farm and the source of the infection was the bite of infected midges.

There have been few epidemiological studies on *Onchocerca* infection in donkeys. Reported prevalence values are 65.38% (21) and 82.98% (13) for *O. cervicalis* in Egypt, 34% for *O. raillieti* in Sudan (10), and 4.26% for *O. reticulata* in again Egypt (13). In another study, only one donkey was examined and found to be infected with *O. cervicalis* (22). No influence of sex on the infection rate has been observed (5, 10, 13). However, a statistically (*p* < 0.05) higher prevalence has been reported for *O. raillieti* (64.3%) in donkeys aged between 7 and 10 years (10) and for *O. cervicalis* (100%) in donkeys older than 15 years (10). Similarly, *Onchocerca* infection has been reported to be more common in older horses rather younger horses (23–25), particularly over 15 years of age (25, 26), as the prevalence of infection increases with age (25). Our detection of *Onchocerca* infection in jennies aged 6–14 years thus appears to be in agreement with the results of previous prevalence studies on donkeys and horses.

The occurrence, distribution, and population density of microfilariae in tissues of *Onchocerca* infected hosts may vary by season. The peak of distribution of *O. cervicalis* microfilariae in ventral-midline skin of 15 naturally infected pony mares was investigated over a 13-month period and was shown to be highest during the spring and lowest in the winter, disappearing in the surface layers of the dermis during the winter months (27). A distinct pattern of distribution was also reported in blood samples from 284 camels (*Camelus dromedarius*) where the highest monthly prevalence of *Onchocerca* microfilariae throughout a period of 14 months was detected in June, disappearing in July to September and February (28). The authors concluded that this is correlated with an adaptation of the parasite to the climate, thus affecting the seasonal distribution of the insect vectors (27), and that environmental conditions may arrest the development of microfilariae or influence their distribution in the host's tissues (28). Similar seasonal variations likely occur in the donkey too in terms of the concentration, distribution, and occurrence of microfilariae in tissues, including the bloodstream. Therefore, our finding of *Onchocerca* microfilariae circulating in the peripheral blood of jennies in late spring (June) is in agreement with the results of other studies (27, 28).

Detection of *Onchocerca* microfilariae in the peripheral blood smear of donkeys or other equids is unusual and thus molecular analysis for confirmatory purposes was needed. In our study, donkeys did not show any dermal or ocular sign that might suggest *Onchocerca* infection at the time of admittance to the VTH. Therefore, skin biopsies (see the Section Discussion below) were not included in the initial diagnostic workup. On the other hand, onchocerciasis diagnosis with skin biopsy is laborious and time consuming for equine practitioners and laboratory technicians, as well as being expensive for owners. Even when *Onchocerca* infection was suspected because of microfilariae morphological features, performing skin biopsies of the ventral midline on at least one of the jennies would have required the specific owner's informed consent. Unfortunately, for this veterinary procedure, the jennies need to be sedated to avoid any risk for the operators and also an extended hospitalization time at the VTH, thus leading to economic losses because of the additional costs and lack of income for the owner of a milk production farm. This means that skin biopsies would never have been permitted by the owner given the lack of any clinical evidence and above all with no apparent economic benefit for him. Consequently, we opted to use molecular analysis rather than skin biopsy to confirm a case of microfilaraemia compatible with presumptive laboratory diagnosis of asymptomatic *Onchocerca* infection. This was despite the fact that a skin biopsy is the standard technique for diagnosing equine onchocerciasis. The concentration of microfilariae that we found in the peripheral blood samples (range 1–31 microfilariae in 2 mL) is likely to be much lower than the actual microfilariae concentration in the tissues of the examined jennies because Knott's test is not the most suitable method to detect microfilariae in *Onchocerca* infected equids. *Onchocerca* microfilariae have a very uneven distribution in the dermis of their hosts (29). The most effective method to detect *Onchocerca* microfilariae and accurately estimate their concentration is based on skin biopsies of the midline of the abdomen, preferably near the umbilical region, both in infected donkeys (5, 10) and horses (15, 30–32). For instance, Hussein and El Sammani (5) reported that the density of *O. raillieti* microfilariae in donkeys ranged from 1,200 to 26,200 per gram of skin in the linea alba along the mid-ventral line and from 1,100 to 16,900 per gram of skin in the region of the wither. In donkeys within the age groups of 1–3, 4–6, and 7–10 years, increasing counts per gram of skin were detected with mean values of 1,083 (range 100–2,400), 1,444 (1,000–2,000), and 2,040 (1,330–9,000) microfilariae, respectively (10). The number of *O. cervicalis* microfilariae per 8 or 6 mm of biopsied skin in ponies and horses ranged from 1 to 21,570 and 8 to 55,600, respectively (31). In other surveys on horses, microfilarial counts ranged from 18 to 42,446 microfilariae/skin snip (16) and a concentration of 19,770 microfilariae/mg was detected (26).

All four *Onchocerca* infected jennies appeared to be healthy and clinically normal at the initial clinical examination. This is in agreement with Hussein and El Sammani (5) and Matov et al. (33) in *O. cervicalis* and *O. raillieti* infected donkeys, respectively. Matov et al. (33) reported that 12 out of 41 healthy donkeys harbored adults of *O. cervicalis* and eight had microfilariae in their eyes. Hussein and El Sammani (5) found that at post-mortem examination, fibrous nodules ranging from 5–10 mm to 2–5 cm in diameter, containing degenerated parasites or filled with caseous and calcified necrotic material, were scattered in the ligamentum nuchae. At histological examination, the authors found that live adult worms induced mild inflammation whereas dead worms elicited an intense inflammatory response with the infiltration of large numbers of lymphocytes, macrophages, and fibroblasts intermingled with eosinophils. Moreover, heavy microfilarial skin densities were detected, as mentioned above, and 5 to 15 microfilariae per eye were found, mostly in the anterior chamber. However, despite these findings, none of the 373 infected donkeys had shown clinical signs that could be attributed to the infection, such as poll-evil and fistulous withers, pruritus, blindness or periodic ophthalmia. Likewise, after surveying over 120 horses infected with *O. cervicalis*, Mellor (34) reported that pathological effects of adult worms on the ligamentum nuchae were of little importance and that no pathological conditions were seen, in either the skin or the eyes, that could be definitely attributed to microfilariae. Matov et al. (33) reported that microfilariae of *O. cervicalis* cannot be considered as the primary cause of periodic ophthalmia in horses. However, other authors have associated *O. cervicalis* infection in horses with a variety of ocular lesions of the conjunctiva, cornea, uveal tract, lens and retina (35), fistulous withers (36), and dermatitis of the ventral midline and/or limbs, shoulders, thorax, and withers (17). It is thought that microfilariae migrate through the bloodstream reaching the eyelids, cornea, conjunctiva, and uvea where they concentrate and that a marked inflammatory reaction is triggered by immune responses to antigens released by dead and dying microfilariae (1). However, the pathogenic role of *O. cervicalis* still remains uncertain and controversial (2, 37) as the infection can be seen in large numbers of horses with or without clinical signs (38). Since we observed the absence of dermatological and ocular signs, we concluded that neither adult worms nor microfilariae appear to cause any clinical evidence in donkeys. It is possible that donkeys may have a higher tolerance than horses to *Onchocerca* infection.

To the best of our knowledge, only two studies have previously investigated hematological values in donkeys naturally infected with *Onchocerca*. The results of one of these studies showed that levels of serum total proteins, albumin, and globulins were significantly (*p* < 0.05) higher compared to uninfected donkeys. Other variations included an increase in the level of glucose and a lower concentration of serum cholesterol (10). By contrast, in jenny I we found low values of serum total proteins and a mild reduction in albumin along with increased cholesterol. However, comparing our findings with those previously reported is not reliable because of the large differences in study design between our report and previous studies. In fact, one study investigated blood biochemical changes following treatment with doramectin against *O. raillietti* microfilariae (9), while another study compared the effects of *O. raillieti* infection on serum total proteins, albumin, glucose, and cholesterol in naturally infected and uninfected donkeys (10). We detected a variety of hematological alterations in one of the jennies (I) with subclinical *Onchocerca* infection. However, all these alterations were probably due to the blood lipemic status secondary to obstructive colic in a lactating jenny and it is very unlikely that *Onchocerca* infection plays a key role in this context. Differential white blood cell counts revealed moderate eosinophilia in jenny II while remarkable eosinophilia and basophilia were present in jenny III. The role of eosinophils in parasitic helminth infections in mammals has been widely demonstrated (39, 40), including in cases of *Onchocerca* infection in humans (41, 42). The relationship between basophilia and helminth infections has also been well established in humans (43) and several animal models (44). However, it cannot be ruled out that these hematological alterations were caused by concurrent infections with other pathogens. Therefore, further investigations are needed to determine whether most donkeys naturally affected by subclinical infection with *Onchocerca* may develop eosinophilia and basophilia.

## Conclusions

*Onchocerca* microfilariae were incidentally detected in four jennies in Italy. Microfilariae were identified by blood smear, Knott's test, and finally DNA analysis. All the cases were of autochthonous origin because the jennies were born and reared on the same farm located in central Italy and they had never moved outside the farm. The transmission of *Onchocerca* infection to these animals is linked to their exposure to bites of infected *Culicoides*, suggesting that vectors in turn could have acquired the infection locally. The reproductive lifespan of *Onchocerca volvulus*, the etiological agent of river blindness in humans, ranges from 9 to 11 years (45) and adult females can release 700 to 900 microfilariae daily (46). At present, the lifespan of *Onchocerca* species in donkeys is unknown but we can assume that adult females live for years and likewise release hundreds of microfilariae daily during their lifespan. Insect vectors that are suitable for transmitting the infection are widespread in Italy (20). Since the infection can be easily overlooked, a high proportion of donkeys may be infected with minimal or no clinical or hematological evidence, and may act as clinically asymptomatic carriers. Our report demonstrates the occurrence of *Onchocerca* in donkeys in Italy. We thus believe that equine practitioners should be aware of our findings and of their implications for equine health. Our findings highlight: (i) the risk of spreading the infection to other susceptible hosts (donkeys, mules, horses, ponies), (ii) the risk of progression in parasitized equines toward clinical disease, and (iii) the need to control the occurrence of *Onchocerca* infections in the breeding systems of donkeys and horses reared in Italy. The possibility of accidental transmission of *O. cervicalis* to humans has also been reported (47, 48). Our report also provides a basis for further studies to determine infection rates in donkeys and horses, to identify the insect vectors that may act as intermediate hosts, and to implement possible prevention and control measures.

## Data Availability Statement

The raw data supporting the conclusions of this article will be made available by the authors, without undue reservation.

## Ethics Statement

Ethical review was not required as per institutional guidelines/local legislation due to natural infections that occurred spontaneously. Written informed consent was obtained from the owners for the participation of their animals in this study.

## Author Contributions

RP performed parasitological examinations and wrote the first draft of the manuscript. GL performed hematological examinations. MS performed all clinical examinations. All the authors critically edited and reviewed the manuscript. All authors contributed to the article and approved the submitted version.

## Conflict of Interest

The authors declare that the research was conducted in the absence of any commercial or financial relationships that could be construed as a potential conflict of interest.
